# Seasonal dynamics and life histories of three sympatric species of *Pseudocalanus* in two Svalbard fjords

**DOI:** 10.1093/plankt/fbab007

**Published:** 2021-03-10

**Authors:** Elizaveta A Ershova, Margot U Nyeggen, Daria A Yurikova, Janne E Søreide

**Affiliations:** Department for Arctic and Marine Biology, Faculty for Biosciences, Fisheries and Economics, UiT The Arctic University of Norway, Tromsø 9037, Norway; Institute of Marine Research, PO Box 1870 Nordnes, Bergen 5817, Norway; Shirshov Institute of Oceanology, Russian Academy of Sciences, 36, Nahimovskiy prospekt, Moscow 117997, Russia; University Centre in Svalbard, Pb. 156, Longyearbyen 9171, Norway; Department of Biological Sciences, University of Bergen, PO Box 7803, Bergen 5020, Norway; Department of Invertebrate Zoology, Faculty of Biology, Lomonosov Moscow State University, Leninskie Gory 1/12 Moscow 119234, Russian Federation; University Centre in Svalbard, Pb. 156, Longyearbyen 9171, Norway

**Keywords:** Copepoda, zooplankton, life cycles, polar regions, species-specific PCR, climate change

## Abstract

Small copepods are the most diverse and numerous group in high-latitude zooplankton, yet our knowledge of important species remains poor because of the difficulties involved in correct species identification. In this study, we use a molecular method of identification, a species-specific polymerase chain reaction, to provide the first description of the seasonal dynamics and life histories of the important genus *Pseudocalanus* in two Svalbard fjords with contrasting environments. We conducted monthly investigations in the relatively warm and ice-free Adventfjorden, supplemented with seasonal samples from the colder, seasonally ice-covered Billefjorden. We found three species of *Pseudocalanus* (the *Arctic P. acuspes* and *P. minutus,* and the *boreal P. moultoni)*. *Pseudocalanus acuspes* had a distinct annual life cycle and dominated during summer, when it actively reproduced. Surprisingly, the boreal *P. moultoni* was present year-round in both fjords and was the dominant species during winter; the presence of all life stages of this species throughout the year suggests a more continuous reproduction. The Arctic *P. minutus* was the rarest of the three species and was likely able to complete its life cycle in Billefjorden but not in Adventfjorden. Our study demonstrates that closely related species may have different life strategies and environmental preferences, which presumably make high-latitude zooplankton communities more resilient to climate change impacts on genus but not necessarily on species level.

## INTRODUCTION

High-latitude pelagic systems are governed by a strong seasonality due to extreme changes in light and thus algal food availability (Leu *et al.*, 2015). Zooplankton grazers living in these regions must tune their life cycles to capitalize on the short, intense primary production period to grow, reproduce, and to build lipid stores that allow them to survive long periods of darkness and low production. The Arctic is rapidly changing, with declining ice cover, rising wave and storm activity, higher sea temperatures and increasing inflow of sub-Arctic water masses into the Arctic (Meredith *et al.*, 2019; Polyakov *et al.*, 2020). Despite the drastic environmental changes, the sun’s activity at high latitudes will continue to create extended periods of continuous light and darkness, limiting light-driven production, which will result in a changed environment for both resident and advected zooplankton species. It is the different species’ ability to quickly adapt to this changing environment, or more specifically, the species’ plasticity and robustness, that will determine the success the individual species and the ecosystem as a whole.

Although year-round observations are still relatively scarce in the Arctic, the life histories and life strategies of zooplankton have been well described for key members of the plankton such as the genus *Calanus* (e.g. Conover, 1988; Slagstad and Tande, 1990; Kosobokova, 1999; Freese *et al.*, 2016), which dominate most Arctic and sub-Arctic zooplankton communities in terms of biomass. However, numerically zooplankton communities are dominated by small copepods, and seasonal cycles of these groups remain poorly studied. One of the most important groups in high-latitude zooplankton is the genus *Pseudocalanus* spp., which in many regions dominates numerically and can account for up to 5–25% of the total biomass (Hopcroft and Kosobokova, 2010; Ershova *et al.*, 2017; Carstensen *et al.*, 2019). The genus is composed of seven species (Frost, 1989), which share a very similar morphology, but are characterized by different, partially overlapping geographical distributions, with several members of the genus frequently co-occurring together in the same area. Two species are common in Arctic shelf seas: *P. acuspes* and *P. minutus*, but sub-Arctic species can also be transported into the Arctic with Pacific and Atlantic water currents. For example, in the Pacific Arctic, ‘warm’ years were shown to be characterized by a high presence of the temperate *P. mimus* and *P. newmani*, while during ‘cold’ years, the two Arctic species *P. acuspes* and *P. minutes* dominated (Ershova *et al.*, 2017). In Svalbard, the Atlantic *P. moultoni*, which is remarkably similar morphologically to *P. acuspes*, was recorded for the first time in 2004 using genetic tools (Aarbakke *et al.*, 2011). The even more temperate *P. elongatus* was recently observed in the north Norwegian Sea, well above the Arctic Circle (Ershova *et al*., submitted), and it is not unexpected that this species will also continue to expand northwards.

Svalbard is a unique region in the Arctic because it experiences typically high-Arctic light conditions, with nearly 4-month periods of continuous light and continuous darkness but is strongly influenced by Atlantic currents, which results in most fjords in western Spitsbergen remaining ice-free throughout the entire winter. In recent decades, extensive ‘Atlantification’ has been documented in Svalbard fjords across multiple trophic levels, with Atlantic taxa replacing their Arctic counterparts (Gluchowska *et al.*, 2016; Vihtakari *et al.*, 2018) The genus *Pseudocalanus*, which contains both Arctic and Atlantic members, can serve as a useful ‘gauge’ of the extent of Atlantification in Arctic systems. However, very few plankton studies identify this group at the species level due to very subtle morphological differences between species, which at the juvenile stages are practically indistinguishable. In recent years, the development of genetic tools has significantly facilitated organism identification, including for the genus *Pseudocalanus* (Grabbert *et al.*, 2010; Aarbakke *et al.*, 2014; Bucklin *et al.*, 2015; Ershova *et al.*, 2017; Ershova, 2020); however, no study thus far has examined the dynamics of these species over a seasonal cycle. In this study, we employ a species-specific polymerase chain reaction (ssPCR) protocol to routinely discriminate between the three species observed in two Svalbard fjords with contrasting oceanographic conditions, and to describe their population dynamics and life cycles over a seasonal cycle for the first time in an Arctic system.

## METHOD

### Study area

**Fig. 1:**
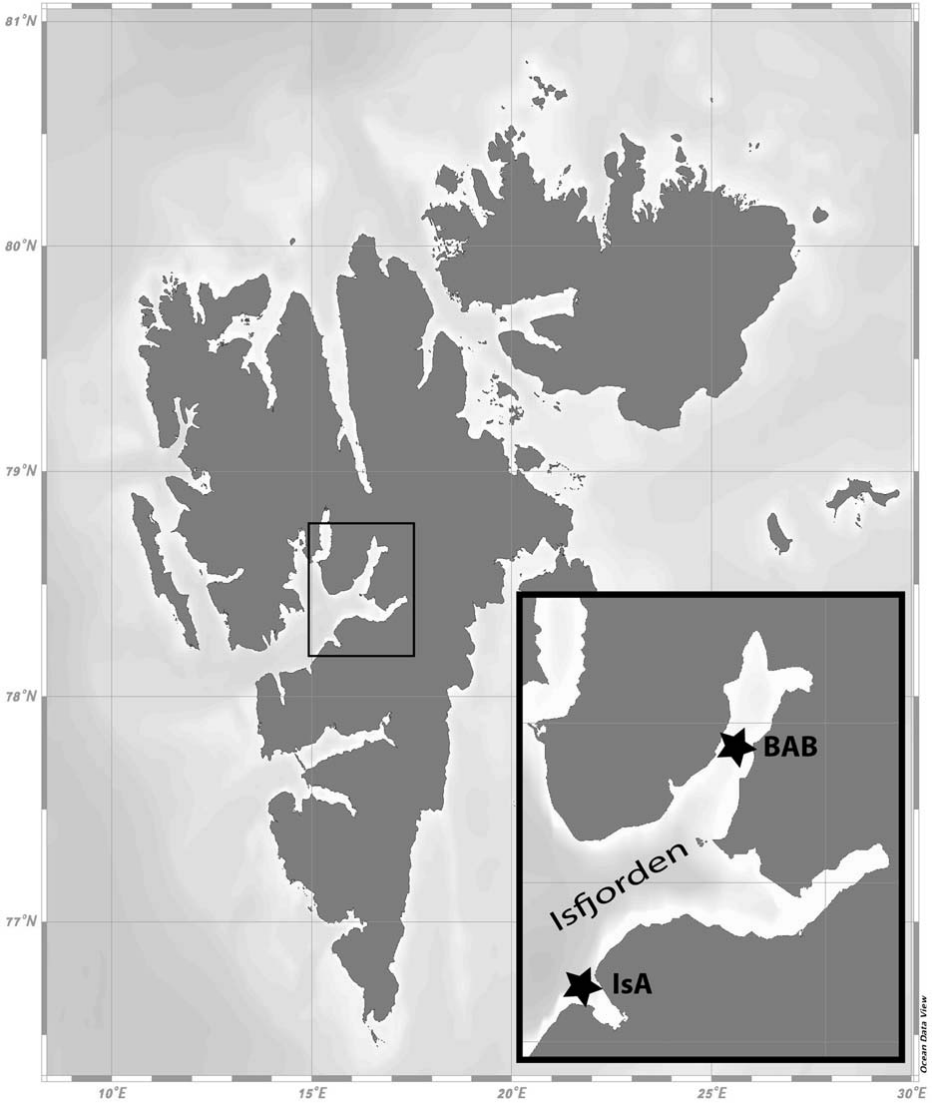
Map of study area, IsA—Isfjorden-Adventfjorden sampling station; BAB—Billefjorden.

The study was conducted in Isfjorden, the largest fjord system on the west coast of Spitsbergen (Fig. 1). Adventfjorden (78.3°N, 15.5°E) is a small side fjord (3.5 km wide and 8 km long) of Isfjorden. This fjord is 60–120 m deep and has no sill. The fjord is mainly influenced by Atlantic water from the West Spitsbergen Current and remains ice-free throughout the winter. Sampling was conducted at the time series station Isfjorden-Adventfjorden (stn. IsA), situated at the fjord mouth at 78.26°N, 15.53°E, from March 2018 to February 2019. The second study site was located in Billefjorden (78°40′N, 16°40′E), another fjord arm in the Isfjorden system where a shallow sill prevent water exchange with the rest of Isfjorden and thus severely restricts advection of warm Atlantic water. Billefjorden is therefore primarily shaped by local processes resembling ‘true’ Arctic environmental conditions, with a pronounced seasonal ice cover from December to June, and biological communities dominated by Arctic species (Arnkværn *et al.*, 2005). Sampling in this location was conducted at the time series station in Billefjorden, Adolf Bukta (stn. BAB) in May 2019 during a period of ice cover, near the ice edge, and in open water in July, August and November 2019.

### Hydrography

Sampling was conducted from larger research vessels (RV Helmer Hanssen and RV Dalnie Zelentsy) and small open boats (Supplementary Table I). The *in situ* conductivity, temperature, density and fluorescence were measured at both stations and all sampling dates (with the exception of fluorescence in IsA during June 2018). A handheld SD208 CTD (SAIV, Bergen) was used at all IsA stations and dates, except at IsA May 11 and August 18 when a SBE911+ CTD-Rosette water sampler system (Sea-Bird Electronics Inc.) was used onboard R/V Helmer Hanssen. The handheld CTD, programmed to make a measurement every second, was lowered to around 10 m above the sea floor with a speed of maximum 0.5 m s^−1^, after leaving it 1 min just below the surface for acclimatization, and was raised with the same speed.

### Zooplankton collection

Mesozooplankton was collected using a WP2 net (Hydro-Bios, Kiel) with a mouth opening of 0.25 m^2^. A mesh size of 60 μm was used for all months, except May 2018 in Adventfjorden during the spring bloom when a 200-μm net was used to avoid clogging of the finer mesh net. All Billefjorden samples were collected using a 200-μm net as part of the regular IMOS (Isfjorden Marine Observatory Svalbard) and Økokyst Svalbard sampling. The net was lowered to approximately 10 m above the seafloor and towed vertically with a speed 0.5 m s^−1^. Minimum two hauls were taken at all stations; one was fixed with 4% buffered formalin and the other with 80–96% ethanol.

### Zooplankton processing

*Pseudocalanus* spp. was counted quantitatively from either the formalin or ethanol samples (Supplementary Table II). Samples were washed from the fixative and diluted with filtered seawater to a fixed volume. Five milliliter subsamples were taken using a 5-mL Finntip pipette until a minimum of 100 *Pseudocalanus* spp. individuals were counted under a Leica Stereomicroscope at 25–40× magnification. Counted individuals were separated by copepodite stage [C1—C5, adult females (AF), adult males (AM)]. Nauplii were also identified and counted, although nauplii counts should not be considered quantitative in the 200-μm mesh nets (e.g. from Billefjorden 2019 and station IsA in May 2018).

### Molecular identification

**Fig. 2:**
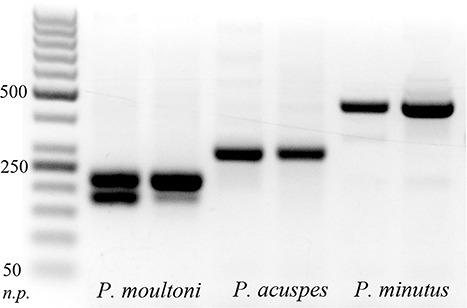
An example of an ssPCR on a 2% agarose gel separating the three *Pseudocalanus* species. n.p.—nucleotide pairs (modified from Ershova, 2020).

Between 30 and 140 individuals (typically 80–100) from each sample were identified genetically. In most samples (see Supplementary Table I for list), prosome length of each processed individual was measured using the ZoopBiom digitizing system (Supplementary Table III) (Roff and Hopcroft, 1986). All prosome length measurements used in this study were thus obtained from ethanol-preserved samples, so if there was shrinkage due to preservation, the bias was consistent between samples. Each specimen was soaked in Milli-Q water for 30 min to remove traces of ethanol, after which DNA was extracted from each specimen using the HotSHOT method (Truett *et al.*, 2000). This extraction method is rapid and inexpensive, requiring only 30 min of incubation time and no DNA cleaning steps. Identification was carried out using a ssPCR designed to separate three species that have been described from this area previously: *P. acuspes*, *P. minutus* and *P. moultoni* (Ershova, 2020). The species-specific primers were designed to attach to locations on the COI gene that were conserved within a species but variable between species, in order to amplify fragments with a size difference of 60–150 base pairs (bp). The primers were selected to have a similar melting temperature (within 3–4°C) and were evaluated for primer dimer formation using the online tool Multiple Primer Analyzer (Thermo Fisher Scientific). Two forward primers (one for *P. moultoni* and one for *P. acuspes* and *P. minutus*) and three reverse primers (one for each species) were selected (Table I). A 10 μL polymerase chain reaction (PCR) was carried out, containing 5 μL of ToughMix polymerase master mix, 0.5 μL of each of the five species-specific primers (two forward and three reverse, Table I), 0.2 μL green dye, 1.8 μL sterile water and 0.5 μL extracted DNA. The PCR protocol was 5 min at 95°C; 35 cycles of 40 s 94°C, 40 s 62°C, 50 s 72°C, 7 min at 72°C. The resulting amplicons were placed on a 2% agarose gel together with a 50 bp ladder; identification was carried out visually based on the length of each fragment (Fig. 2). A minimum of 10 and a maximum of 30 individuals of each stage were identified in this way (when present in the sample in sufficient numbers), with a total of 1366 individuals for all samples combined. Although the possibility remains that *P. elongatus* was also present in the samples, a simultaneously conducted metabarcoding study did not detect *P. elongatus* or other *Pseudocalanus* species than the three mentioned above in the Svalbard fjords (Ershova *et al*., submitted). For this reason, and due to the fact that a large portion of the analysis for this study has been carried out prior to the development of species-specific primers for *P. elongatus*, we did not include this species in the identification protocol.

**Table I:** List of primers used, from Ershova (2020)

Species	Name	Length of fragment (base pairs)	Sequence
*P. acuspes/P. minutus* (forward)	PseudoF-mod	-	5′-TTCGAATASARYTRGGHMVRGY-3′
*P. acuspes* (reverse)	acuspes280R	280	5′-AGAGGAGGGTATACAGTTCACC-3′
*P. minutus* (reverse)	minutus480R	480	5′-CGCAAACARAGGTATTTGGTCT-3′
*P. moultoni* (forward)	moultoni307F	-	5′-GCATGCAGGAGGTTCTGTTG-3′
*P. moultoni* (reverse)	moultoni520R	213	5′-ACAATATTGTAATTGCMCCAGC-3′

### Data processing

CTD measurements were quality controlled and interpolated at 1-m intervals. Salinity at each depth was calculated from conductivity using the package oce in R (Kelley, 2018). Water mass types were assigned at 10-m intervals following temperature/salinity thresholds defined in Svendsen *et al.* (2002) and Nilsen *et al.* (2008).

The relative species proportions at each developmental stage, as determined by ssPCR, were multiplied by the quantitative counts of copepodite stages to estimate abundance and population structure of each species per sample. Biomass in dry weight (micrograms) was estimated from prosome length (micrometers) using a regression relationship described for this genus (log_10_DW = −7.62 + 2.85^*^log_10_PL) (Liu and Hopcroft, 2008). Biomass at each station was estimated by multiplying mean weight of that species/stage with its abundance. As zooplankton experiences some shrinking in ethanol, these values may be slightly underestimated. For those samples where no measurements were taken, the average weight of each species/stage from all the samples was used to estimate biomass. Mean developmental stage of each species at each sampling date was calculated by multiplying the proportion (0–1) of each stage by 0–6 (0, nauplii; 1–5, C1—C5 stages; 6, AF/AM) and summing up the values for each stage together. For example, a population that consisted of 20% C1 stage, 70% C2 stage and 10% AF would have a mean stage of 2.2 (0.2^*^1 + 0.7^*^2 + 0.1^*^6).

The correlations between species distribution and population structure of the three *Pseudocalanus* species and the physical environment were investigated using canonical correspondence analysis (CCA). The CCAs were performed on square-root transformed abundance data and scaled physical parameters using the R package vegan (Oksanen *et al.*, 2019). Each species was divided into four developmental stage groups: nauplii–C2, C3–C4, C5 and AF/AM. Examined physical variables included surface, bottom and integrated temperature and salinity, as well as maximum and depth-integrated fluorescence. The best model was selected via the ordistep function in the package vegan in both directions with 10 000 permutations (Blanchet *et al.*, 2008). The significance of the resulting model and predictors were tested, with significance level set to *P* ≤ 0.05. Differences in body size of individuals of the same species/stage between months were tested using a one-way analysis of variance (ANOVA).

## RESULTS

### Physical environment

**Fig. 3:**
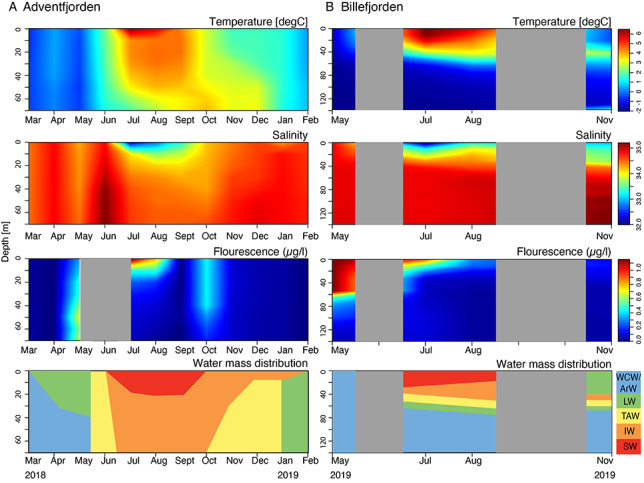
Seasonal trends in temperature, salinity, fluorescence and water mass distribution in (**A**) Isfjorden-Adventfjorden (IsA) during March 2018 to February 2019 and (**B**) Billefjorden (BAB) during May 2019 to November 2019. Water mass abbreviations: WCW/ArW—Winter Cold Water/Arctic Water; LW—Local water; TAW—Transformed Atlantic Water; IW—Intermediate Water; SW—Surface Water.

The water masses in Adventfjorden (Stn. IsA) comprised a mixture of local water (LW), locally formed winter cold water (WCW) and Arctic water (ArW) (as defined in Svendsen *et al.*, 2002; Nilsen *et al.*, 2008) from March to May, but never reached temperatures lower than −0.5°C (Fig. 3). In June, a sharp shift in water mass properties was seen, with warmer (1–2°C) and more saline transformed Atlantic water (TAW) appearing in the entire water column. Snow melt and increased river run off combined with solar heating led to a distinct upper fresher and warmer (up to 7°C) surface water (SW) layer from July to September, with a mixture of SW and TAW below defined as intermediate water (IW). In October, the IW filled the entire water column. In November, TAW appeared below 30 m and filled nearly the entire water column by January 2019. By February, cooler LW dominated.

In Billefjorden, sea ice was still present in May 2019, and WCW dominated with low temperatures between −1.6 and −1.8°C in the entire water column. In July and August, SW with relatively warm temperatures (>6°C) and relatively low salinity (<32) was found in the upper 15–30 m, while below 50 m (and the threshold depth), WCW with temperatures between −1.8 and −1°C still prevailed. In the period between September and November, cooling of the SW and winter convection led to gradually colder SW, while a warmer layer between 35 and 70 m depth remained present.

In Adventfjorden, the spring bloom began in May, with high chlorophyll found throughout the entire water column. Elevated chlorophyll values continued in the surface layers until July, when the Chl-*a* maximum occupied the upper 20 m. A second, smaller bloom appeared in October. In Billefjorden, an intense bloom was observed in May; measurable fluorescence was maintained through July, but in August and in November, estimated chlorophyll values approached zero (Fig. 3).

### Species-specific PCR

Of the 1366 PCR reactions, 1274 (~93%) resulted in successful and unambiguous identification (Supplementary Table II). Eighty individuals produced no bands on the gel and 12 had double banding, presumably from cross-well contamination. About 681 individuals (54%) were identified as *P. acuspes*, 483 (38%) as *P. moultoni* and 110 (9%) as *P. minutus*.

### Abundance and biomass

Overall *Pseudocalanus* abundance ranged from 20 to 570 ind m^−3^, with the highest value observed in Adventfjorden in September and the lowest values in Billefjorden in April and Adventfjorden in May. Although *Pseudocalanus* abundance in Adventfjorden tended to be low during winter/spring from December to June, and generally higher during the summer/fall months (July to November), there was significant month-to-month variability. For example, March was characterized by relatively high *Pseudocalanus* abundance (190 ind m^−3^), comparable to summer values, while abundance in October was much lower than that observed during both September and November. The overall abundance of *Pseudocalanus* in Billefjorden was very low in May (20 ind m^−3^), but by July, increased to 230 ind m^−3^, and remained high during August and November. Overall, abundance was higher in Billefjorden than in Adventfjorden during all months except May. The overall biomass ranged between 0.1 and 1.6 mg DW m^−3^ and, contrary to abundance trends, was highest in Billefjorden during August and November (Fig. 3B). Overall, biomass values were less variable month-to-month than abundance with less distinct seasonal peaks and no pronounced decline in biomass observed during winter.

**Fig. 4:**
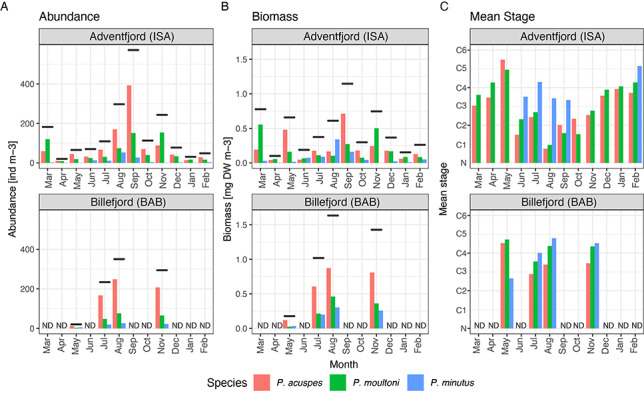
Abundance (**A**) and biomass (**B**) of three *Pseudocalanus* species in Isfjorden-Adventfjorden (IsA) during March 2018 to February 2019 and Billefjorden during May 2019 to November 2019, horizontal bars indicate total abundance/biomass of the genus; (**C**) mean developmental stage of the population; C1–C6—copepodite Stages 1 to adult; N—nauplii. Shown only for stations where a minimum of 10 individuals of that species were identified. ND—no data.

All three *Pseudocalanus* species co-occurred in both fjord locations (Stns IsA and BaB) at most sampling dates, but the relative species composition varied between the two locations and between seasons (Fig. 4A). *Pseudocalanus acuspes* was the dominant species in Adventfjorden from May to October, reaching up to 400 ind m^−3^ in September. However, abundance of this species dropped dramatically during the winter months, reaching a minimum in April (10–50 ind m^−3^). *Pseudocalanus moultoni* in Adventfjorden had less pronounced seasonal dynamics in abundance, ranging from 10 to 150 ind m^−3^, with higher month-to-month variability and peak numbers observed during March, September and November. During the winter months (November to April), the relative contribution of *P. moultoni* was particularly high, comprising 45–65% of the total *Pseudocalanus* abundance. *Pseudocalanus minutus* was only present in Adventfjorden in measurable quantities during the summer months, from June to September, but the abundance was low (<50 ind m^−3^). During the other months, this species was detected as single individuals and represented less than 1% of total *Pseudocalanus* numbers. In Billefjorden, *P. acuspes* was the dominant species during all sampling months. *Pseudocalanus moultoni* was present in much lower numbers than in Adventfjorden, with the lowest values (<3 ind m^−3^) observed in May. In July to November, however, it was present in significant quantities (50–75 ind m^−3^), comprising 20–25% of the total *Pseudocalanus* abundance. Similar to Adventfjorden, *P. minutus* was the least abundant species in Billefjorden, but unlike the former, it was observed in Billefjorden during all sampling months (~20 ind m^−3^). Biomass of the three species generally followed slightly different trends from those of abundance, accounting for the larger body sizes of older developmental stages. As such, *P. acuspes* had biomass peaks in May and September, while *P. moultoni* peaked in March and November. In terms of biomass, the larger-bodied *P. minutus* played a relatively larger role at both locations and dominated the biomass in Adventfjorden in June and August.

### Population structure

For *P. acuspes*, the distribution of developmental stages displayed a strong seasonality in Adventfjorden, with nauplii and C1–C2 stages appearing from June to November and being absent during the other months (note that the May sample was collected with a 200-μm net, so nauplii may have appeared in the plankton earlier) (Figs 4C and5A). A distinct shift from a predominance of late life stages occurred between May and June (going down from mean stage 5.5 to 1.5) and the population comprised primarily of early life stages C1–C3 until November. During the winter months (December to April), almost the entire population was composed of C3–C4 stages (mean stage 3.8). Stage C5 peaked in abundance in May, and during the remaining months of the year, C5 copepodites were present in very low numbers (<3%). AF showed a strong abundance peak in May and re-appeared in the plankton again in August to September. During the other months, females of *P. acuspes* were practically absent. AM were observed in May and July. In Billefjorden, *P. acuspes* had an even more pronounced seasonal cycle than in Adventfjorden, with nauplii appearing in May and absent during the other months. C1 stages appeared in July and disappeared by August, with a clear succession of the population to the overwintering stages C3–C4. In contrast with Adventfjorden, however, AF and AM were present during all months, except November when no males were observed.

**Fig. 5:**
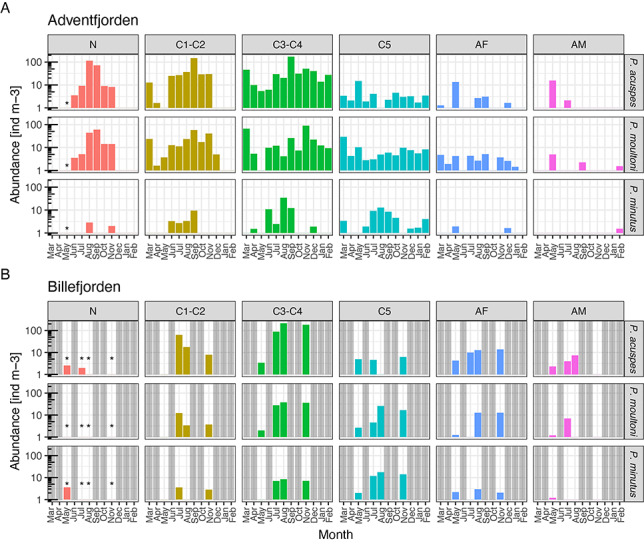
Population structure and abundance of developmental stages across the seasonal cycle in (**A**) Adventfjorden and (**B**) Billefjorden. C1–C5 copepodite Stages 1–5; N—nauplii; AF—adult females; AM—adult males. Note that abundances are shown on a logarithmic scale. Gray bars indicate no data.

For *P. moultoni*, a shift from older stages to younger stages was also apparent in May in Adventfjorden, but the drop was less pronounced (from 4.9 to 2.2) than in *P. acuspes* (Figs 4C and5A). Although nauplii were detected only during the summer months, just as *P. acuspes*, young copepodite stages of *P. moultoni* (C1–C2) were also present in low numbers (<10 ind m^−3^) during several of the winter months. C5 copepodites and AF of this species were relatively numerous (>10 ind m^−3^) year-round, and the overwintering population was composed of stages C3 through adult. In Billefjorden, no nauplii of *P. moultoni* were observed, but it is likely that they merely failed to be captured with the 200-μm net, since the overall abundance of this species was low and life stages C2–C5 were recorded during all months. In Billefjorden, the presence of *P. moultoni* AF was extremely low in May and not detected in July, but their numbers increased substantially in August and November (Fig. 5B).

The presence of *P. minutus* in Adventfjorden was only confirmed between June and August, and they comprised almost entirely of later developmental stages with only a few nauplii and C1’s recorded (<0.5 ind m^−3^). In Billefjorden, nauplii of *P. minutus* appeared in May and C1 stage in July.

### Body length of adult females

**Fig. 6:**
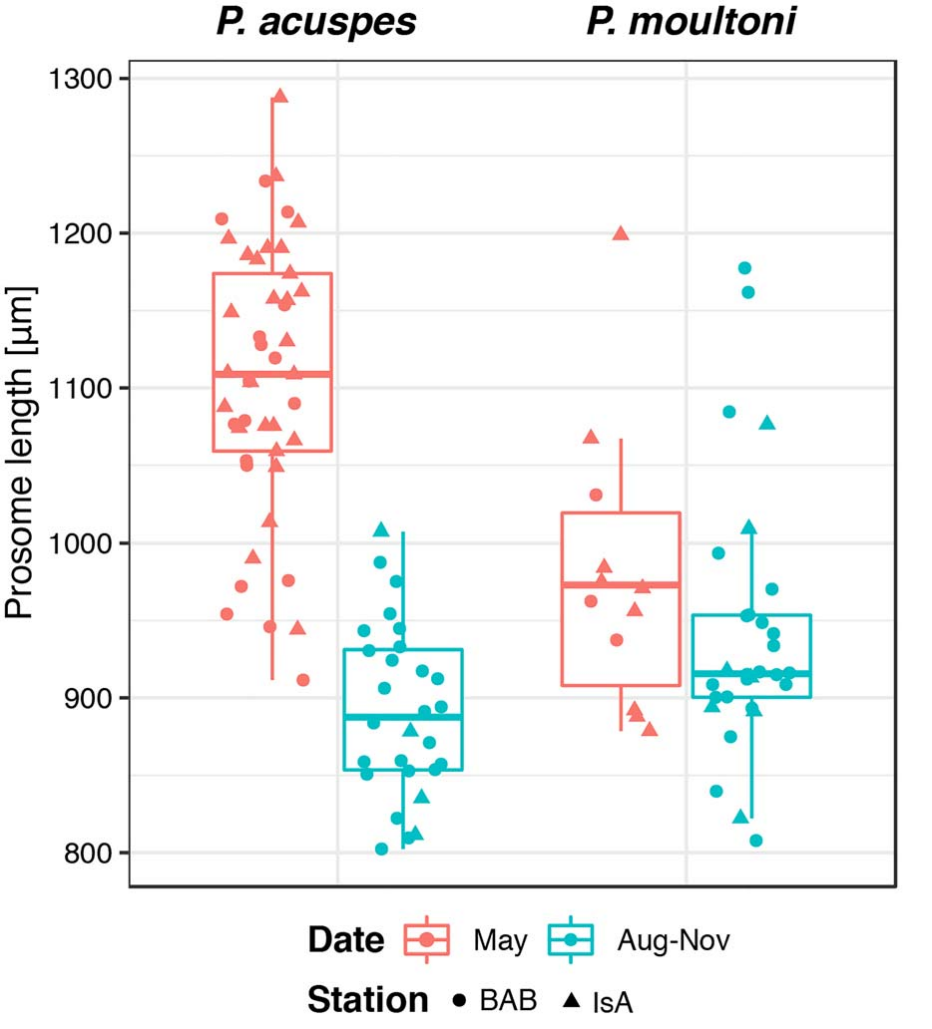
Prosome length of adult females of *P. acuspes* (left) and *P. moultoni* (right) in May vs. August to November. No data are presented for *P. minutus* and for other months due to low numbers of individuals observed.

The body size of adult females (AF) of *P. acuspes* varied among months, with individuals observed in May in both locations being distinctly larger (by about 200 μm) than those observed during late summer–fall (ANOVA, *P* < 0.01) (Fig. 6). The late-summer females of *P. acuspes* were the same size or even smaller than the typically smaller-bodied *P. moultoni*. For *P. moultoni*, the females were on average slightly smaller in late summer to autumn than in spring, but the differences were much less pronounced and not statistically significant.

### Relationship between population structure and physical parameters

**Fig. 7:**
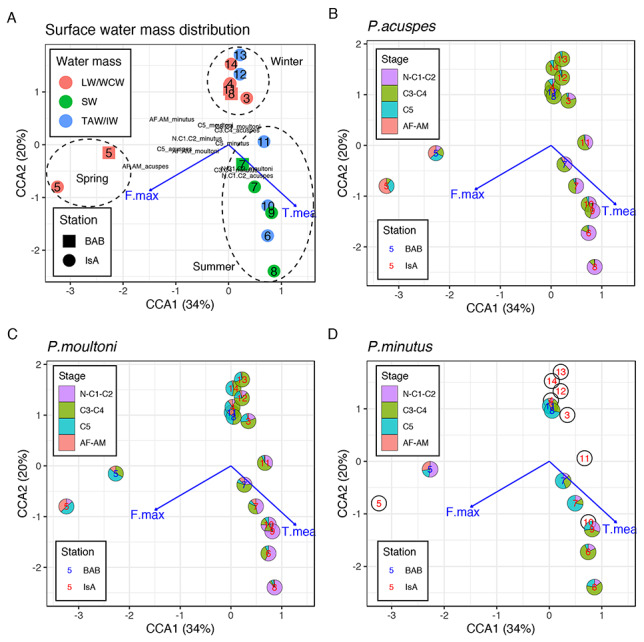
CCA ordination diagram for square-root transformed abundance of *Pseudocalanus* species/developmental stages and environmental variables in Billefjorden and Adventfjorden with overlaid (**A**) water mass type, (**B**) stage composition of *P. acuspes*, (**C**) stage composition of *P. moultoni* and (**D**) stage composition of *P. minutus*. Empty circles indicate absence of sufficient data to evaluate stage composition. Numbers indicate sampling months; text color in B–D indicates location: blue—Billefjorden; red—Adventfjorden. T.mean—mean temperature; F.max—maximum fluorescence. Dashed circles delineate the winter, summer and spring groups. IsA—Isfjorden; BAB—Billefjorden; numbers after the station name indicate sampling month.

The best CCA model included mean temperature (*P* = 0.01) and maximum fluorescence (*P* = 0.03) as significant predictors for species and stage composition (Fig. 7). The model accounted for 54% of total inertia in the data, with the first two axes accounting for 83% of the explained variance. The ordination showed a clear seasonal pattern, with a distinct Adventfjorden winter group (November to April), dominated by *P. acuspes* C3–C4 and *P. moultoni* C3–C5 stages. Interestingly, August and November in Billefjorden were characterized by very similar species composition and hydrology and grouped with the Adventfjorden winter group. A distinct summer group included both fjord stations between July and October and was associated with warmer water and was dominated by early life stages of *P. acuspes* and *P. moultoni* and sub-adults of *P. minutus.* The third group, which was the most distant from the rest, included May samples from both fjords, which were distinguished by high fluorescence values and high abundances of adults of *P. acuspes* and scarcity of other developmental stages.

## DISCUSSION

### Pseudocalanus distribution in Svalbard fjords

Although most zooplankton studies carried out in Svalbard recognize *Pseudocalanus* as an important component of the zooplankton community (Weslawski *et al.*, 1991; Walkusz *et al*., 2003, 2009; Daase and Eiane, 2007; Weydmann *et al.*, 2013; Gluchowska *et al.*, 2016; Hop *et al*., 2019a, b), these studies generally list the genus as a single group without attempting to differentiate between species. The few studies that describe *Pseudocalanus* at the species level have only focused on AF (Aarbakke *et al.*, 2011, 2017), or merely made the assumption that most the individuals belonged to one species (Lischka and Hagen, 2005). Nonetheless, some results of these earlier studies describe broad patterns that are comparable to the present work. The highest abundances of this genus have been typically observed in the more ‘Arctic-type’ fjords (Cottier *et al.*, 2010) in western (Hornsund, Weslawski *et al.,* 1991), northern (Rijpfjorden, Weydmann *et al.*, 2013) and eastern Svalbard (Storfjorden, Werner, 2005; Hirche and Kosobokova, 2011). This is also the case in our study, with the higher abundance of *Pseudocalanus* observed in the Arctic-type Billefjorden than in the warmer Atlantic-influenced Adventfjorden during all months except May. This becomes even more apparent when looking at the absolute values (per unit area), which were nearly three times higher in the deeper Billefjorden (190 m) than in the shallow Adventfjorden (70–90 m). Adventfjorden is ice free with water temperatures above freezing year-round during most years, resulting in more boreal conditions for the fauna living there. Advection of Atlantic water into the west coast Svalbard fjords occurs primarily during the summer months, and during winter, they generally contain locally formed winter water (Cottier *et al*., 2010), but wind-driven advective events can also occur in winter, causing upwelling of warm Atlantic water onto the West Spitzbergen shelves and into the fjords (Skogseth *et al.*, 2020). The relatively warm temperatures that we observed in Adventfjorden during March to April 2018 (>0°C) together with high month-to-month variability in *Pseudocalanus* abundance—e.g. the anomalously high abundance of *Pseudocalanus* observed during March—may have been due to such advective event(s). Billefjorden is less influenced by advection, resulting in more stable environmental conditions and less month-to-month variability in the zooplankton communities. The very low abundances of *Pseudocalanus* that we observed in May, while the fjord was still ice-covered, likely reflects an annual ‘reset’ of the populations, marking the onset of a new generation (see section on life cycles below).

Previous studies that used genetic methods for identification have found *P. minutus* to be the dominant species in a number of Svalbard fjords, including Kongsfjorden (Lischka and Hagen, 2005) and Billefjorden (Aarbakke *et al.*, 2011, 2017). However, these studies generally identified only AF, and our work strongly highlights the importance of taking into account the entire population. Fox example, if we had included only AF in our analysis, we would have come to the erroneous conclusion that *P. moultoni* was the only species present in Adventfjorden during the majority of the year. When the entire population was accounted for, *P. acuspes* was found to be the dominant *Pseudocalanus* species in both fjords, and *P. minutus* the least abundant, which is a somewhat surprising result, given previous records. The fact that earlier works focused only on AF explains, however, only part of the discrepancy, as the highest proportion of AF in our study was maximum 25% for *P. minutus*, in Billefjorden during May. In Adventfjorden and during other months in Billefjorden, its contribution was even lower, with generally <5–10% females belonging to *P. minutus*. Elsewhere, *P. minutus* dominates in seasonally ice-covered Arctic shelf seas (Melnikov *et al.*, 2005; Persson *et al.*, 2012; Ershova and Kosobokova, 2019), including waters around Svalbard (Werner, 2005; Weydmann *et al.,* 2013), but it is also numerous in the deep waters of the North Atlantic to the east of Jan Mayen (Wiborg, 1955; Aarbakke *et al.*, 2017). This is an interesting example how the same species can occupy two very different habitat types, and it remains to be determined whether they represent isolated populations, subspecies or even two cryptic species. In general, *P. minutus* seems to prefer deeper, colder waters than the other *Pseudocalanus* species (Ershova *et al.*, 2016), which would explain its low numbers in Adventfjorden. The low numbers that we observed in Billefjorden compared to previous studies could be due to our less frequent sampling here and thus missing the time window when this species peaks in abundance—at least for AF, which previously have been found to peak in abundance during March to May (Weydmann *et al*., 2013) and in Billefjorden during March (Aarbakke *et al.*, 2017). However, it could also be due to improper identification in the earlier works or due to recent possible climate-induced shifts towards more boreal species (see below).

Another unexpected result of our study was the year-round presence of the boreal *P. moultoni* in both examined fjords, where it composed up to 65% of the bulk *Pseudocalanus* abundance for several months of the year. Historically, *P. moultoni* was likely misidentified as *P. acuspes*, as these two species are virtually indistinguishable morphologically (Frost, 1989), and only *P. acuspes* was known to occur in the European Arctic (Frost, 1989). Genetically, however, these two species are among the most divergent *Pseudocalanus* species (Aarbakke *et al.*, 2017). The distribution of *P. moultoni* was recently described using molecular identification, but these studies were only based on AF abundances (Aarbakke *et al.*, 2014, 2017). *Pseudocalanus moultoni* has been observed in the North Atlantic Ocean, along the Norwegian coast, and in several fjords in Svalbard, including ‘Arctic’ ones such as the seasonally ice-covered Rijpfjorden and Billefjorden (Aarbakke *et al.*, 2017). From these studies, it was unclear if *P. moultoni* was an advected Atlantic expatriate, such as *Oithona atlantica* or *Limacina retroversa*, or if Svalbard was part of its central distribution range. Only a few details are known about the ecology of *P. moultoni*, and those were mainly described prior to the development of molecular tools and thus before correct species identification could be proved (e.g. McLaren *et al.*, 1989).

### Life history strategies of *Pseudocalanus* in Svalbard

Arctic marine organisms have evolved life cycles adapted to the extreme seasonal environment in Polar regions (Hagen and Auel, 2001), which also determine prey availability for higher trophic levels. Life cycles of Arctic zooplankton have been well described for the genus *Calanus* (e.g. Falk-Petersen *et al.*, 2009 and references therein), but small copepods, such as the numerous genus *Pseudocalanus*, have so far received little attention. Most *Pseudocalanus* life cycles were described before it was recognized that multiple species co-occur in the same Arctic and sub-Arctic locations (Pertsova, 1981; McLaren *et al.*, 1989; Norrbin, 1991; Lischka and Hagen, 2005). Particularly the early life stages were easily misidentified, which may have skewed the results of these earlier works. The few recent studies that have used genetics to discriminate between species (Aarbakke *et al.*, 2014, 2017; Bucklin *et al.*, 2015) have so far focused on AF only, which during most times of the year represent less than 5% of the population, or were studies restricted to single seasons (Ershova *et al.*, 2017) not taking into account seasonal dynamics of the populations.

**Fig. 8:**
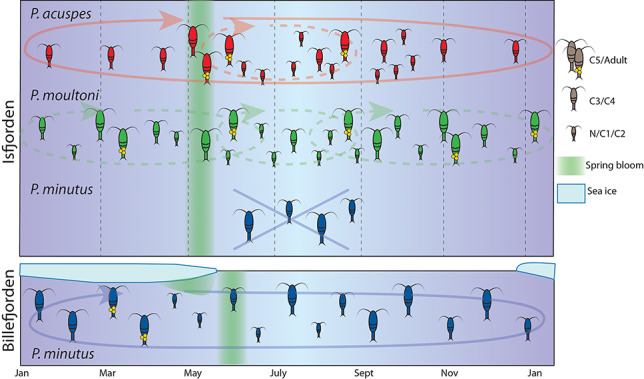
Conceptual model of the life histories of *Pseudocalanus acuspes*, *P. moultoni* and *P. minutus* in Adventfjorden and *P. minutus* in Billefjorden. *Pseudocalanus acuspes* had a distinct annual cycle, with peak reproduction observed during June to September, and a second, smaller generation appearing at the end of the summer period. *Pseudocalanus moultoni* had less pronounced peaks in abundance and stage composition and is presumed to have a more continuous reproduction, with an unknown number of generations per year. *Pseudocalanus minutus* presumably has an annual cycle in Billefjorden, timing its reproduction to the ice bloom, but does not complete its life cycle in Adventfjorden.

Polar copepod species generally differ from their low-latitude counterparts in that they synchronize their reproduction to take advantage of the brief spurts of growth during the short Arctic summer, resulting in distinct cohorts, or generations, appearing in sequence. Species from lower latitudes, on the other hand, often exhibit continuous reproduction, such that all developmental stages are present in the plankton simultaneously. Of the three examined *Pseudocalanus* species, the life cycle of *P. acuspes* was the most typical for the Arctic (Fig. 8). Based on the seasonal dynamics of life stages, this species had a distinctive annual cycle in both sampling locations with a shift in generations in May to June and a prolonged period of reproduction between May and November. The earliest recruits produced in spring likely reached adulthood and maturity by August to September, resulting in a second summer generation, which explains the distinctly smaller females observed during that time compared to spring. Their offspring, as well as the remainder of the spring population, overwintered at the C3–C4 stages, suggesting that these are dedicated resting stages and that this species will not molt further in the absence of abundant food. During the peak phytoplankton production in May, these C3–C4 stages rapidly developed through the C5 stage and reached maturity shown as a distinct increase in adults in May after the onset of the spring bloom with first appearance of nauplii also occurring at this time.

Our results demonstrate that *P. moultoni* successfully overwinters in Adventfjorden and is able to complete its life cycle there. Although its overall dynamics in stage composition resembled that of *P. acuspes*, there were some notable differences. Unlike *P. acuspes*, *P. moultoni* had no pronounced seasonal abundance peaks in Adventfjorden and reached its highest abundance in the winter months, when abundance of *P. acuspes* dropped dramatically. Although reproduction of *P. moultoni* peaked during the summer, similar to *P. acuspes*, the nearly year-round presence of both early (C1–C3) and late (C5–AF) stages, including AM, suggest more continuous reproduction, in line with a more ‘temperate’ life history strategy (Fig. 8). The lack of a dominant overwintering stage combined with the presence of young life stages year-round suggests that this species is less seasonally restricted than the other two *Pseudocalanus* species. Similarly, the absence of change in body size of females in spring versus summer–autumn also indicates a less seasonally dependent reproductive strategy. It is likely that the unique ‘temperate’ conditions in Isfjorden allow this species to thrive there. Interestingly, although present in much lower numbers, this species was also important in the Arctic Billefjorden. Their very low numbers in May relative to the other two species, however, suggests low survival over the winter, but by early autumn, their stocks are replenished either through local production or advection.

*Pseudocalanus minutus* was only observed in Adventfjorden as late stage (C4—C5) individuals, suggesting that this species does not complete its life cycle within this fjord (Fig. 8). The presence of this species coincided with the inflow of modified Atlantic water, so it is likely that it was carried there with currents from the deeper Atlantic waters, representing the deep Atlantic part, and not the Arctic part of this species distribution range (Wiborg, 1955; Aarbakke *et al*., 2017). The simultaneous presence of another indicator of deep Atlantic water masses, O. atlantica (Nyeggen, 2019), further supports advection to play a major role here. The only location where *P. minutus* nauplii were observed in significant numbers, indicating recent reproduction, was in Billefjorden in May, near the ice edge. Previous studies reported aggregations of *P. minutus* under the Arctic sea ice and evidence of direct feeding on ice algae (Conover *et al.*, 1986; Runge and Ingram, 1991), suggesting that this is an important adaptation of this species to life in the Arctic. *Pseudocalanus minutus* reaches the largest body size of the examined species and has very efficient lipid turnover (Boissonnot *et al.*, 2016), comparable to that of *Calanus*, with more than half of lipids deposited as wax esters. Although our dataset for Billefjorden includes only 4 months and does not cover the winter period, we deduct that this species also has a distinct annual cycle, with a shorter and earlier reproduction period than *P. acuspes*, fueled by ice algae or lipid reserves. Aarbakke *et al.* (2017) reported that 95% of AF of *P. minutus* found in March in Billefjorden belonged to *P. minutus*. Similarly, peak abundances of *P. minutus* females were observed in Rijpfjorden during the spring transition period, under sea ice containing high algal biomass (Weydmann *et al.*, 2013). This likely represents the period when maturity is reached and reproduction takes place, resulting in a peak of nauplii ~45 days later, in May. This allows the early life stages to take advantage of the later spring phytoplankton bloom and reach the main diapausing stages (C4–C5) earlier in the summer, as supported by a dominance of late stages during the remainder of the year (Fig. 8). As such, *P. minutus* is likely to be a capital breeder, employing a very different strategy than *P. acuspes* and *P. moultoni*, which reproduce opportunistically as food becomes available and can continue to reproduce throughout the season.

### *Pseudocalanus* in a future Arctic

Svalbard has experienced dramatic changes in recent decades, with increasing influence of Atlantic water in the fjords carrying sub-Arctic fauna and with many previously ice-covered fjords remaining ice-free throughout the winter (Muckenhuber *et al.*, 2016; Hop *et al.*, 2019b). There is increasing evidence of communities across all trophic levels shifting from an Arctic to a more Atlantic character (Gluchowska *et al.*, 2017; Vihtakari *et al.*, 2018; Hop *et al.*, 2019b). Within the zooplankton, recent shifts in species composition were observed in Kongsfjorden within the genus *Calanus* from the Arctic *C. glacialis* to the temperate *C. finmarchicus* (Hop *et al.*, 2019b). It is fully expected that similar changes will occur, or have already occurred, for *Pseudocalanus* spp., which also contains both Arctic and temperate members. For example, the low numbers of *P. minutus* observed during our study, despite this species being reported as the dominant one in Svalbard previously, may represent anecdotal evidence that a species shift may already have taken place. *Pseudocalanus minutus* thrives in seasonally ice-covered waters, timing its reproduction to sea ice algae blooms (Norrbin, 1991; Weydmann *et al.*, 2013), and several fjords on the west coast of Svalbard have been transitioning from more ‘Arctic type’ to more ‘Atlantic type’, without a winter ice cover (Cottier *et al.*, 2010; Hop *et al.*, 2019b). This may result in a decline of this species. However, this result may also have been due to inaccurate species identification in previous studies. Similarly, the higher contribution of the temperate *P. moultoni* than reported by previous studies may represent an increase in this species’ relative importance or simply being a result of an increased effort in proper molecular identification. Regardless, this study provides an important baseline to monitor the absolute and relative contribution of these species in the future. Additionally, this study did not include species-specific primers for *P. elongatus*, another North Atlantic species that has not yet been recorded in Svalbard or the Arctic but that can also be potentially advected from temporal regions. We cannot exclude that some of the failed PCRs in our study belonged to this species, and this will be up to future studies to resolve.

The coexistence of three very similar species with slightly different life histories and environmental tolerances ensures that despite possible short- or long-term shifts in their relative numbers, e.g. if *P. minutus* are replaced with *P. moultoni*, the overall abundance and productivity of the genus may largely remain the same, ensuring their availability for higher trophic levels. Although *P. minutus* is larger and more lipid rich than the other species (Boissonnot *et al.*, 2016), these differences in energy content may be offset by higher growth and turnover rates of the smaller species, as is predicted for the *Calanus* complex, with *C. finmarchicus* replacing the larger, more lipid-rich *C. glacialis* (Renaud *et al.*, 2018). In fact, these authors predict that the energy transfer between trophic levels will become *more* efficient under these future scenarios. As such, the coexistence of the *Pseudocalanus* species complex similarly represents a mechanism of resilience of Arctic marine systems to climate change.

## CONCLUSION

Our study highlights the importance of seasonal, species-specific investigations that focus on the entire population of an organism spanning an entire seasonal cycle, not just their adult stage. This can lead to very different conclusions, as evidenced by previous studies that found *P. minutus* to be the dominant species in Svalbard. Furthermore, we demonstrate that closely related species can differ significantly in their life cycle strategies and contribution to the biomass of the ecosystem. *P. acuspes* had a distinct annual life cycle in Isfjorden, being the dominant species during the summer months and peaking in reproductive activity and the presence of early life stages. Surprisingly, the boreal *P. moultoni* was present year-round in both fjords and was equally important or even the most numerous *Pseudocalanus* species in winter. This species had a more even distribution of life stages throughout the year, suggesting it to have a continuous reproduction. The Arctic *P. minutus* was the least abundant of the three species in both fjords. It was able to complete its life cycle in the Arctic Billefjorden but not in the Atlantic-influenced Adventfjorden. Since the different species have different environmental adaptations, further climate-related changes in the Arctic may shift their distributions and relative importance in zooplankton communities. The co-occurrence of several closely related species, however, can compensate in the individual species population success, thus ensuring high-latitude zooplankton communities to be more resilient to climate change impacts at the genus level but not necessarily at the species level.

## Supplementary Material

Supplementary_table_1_fbab007

Supplementary_table_2_fbab007

Supplementary_table_3_fbab007

## Notes

Corresponding editor: Marja Koski
